# Emphysematous change with scleroderma-associated interstitial lung disease: the potential contribution of vasculopathy?

**DOI:** 10.1186/s12890-018-0591-y

**Published:** 2018-01-30

**Authors:** Hideaki Yamakawa, Tamiko Takemura, Tae Iwasawa, Yumie Yamanaka, Satoshi Ikeda, Akimasa Sekine, Hideya Kitamura, Tomohisa Baba, Shinichiro Iso, Koji Okudela, Kazuyoshi Kuwano, Takashi Ogura

**Affiliations:** 1 Department of Respiratory Medicine, Kanagawa Cardiovascular and Respiratory Center, 6-16-1 Tomioka-higashi, Kanazawa-ku, Yokohama, 236-0051 Japan; 2 Department of Pathology, Japanese Red Cross Medical Center, Tokyo, Japan; 3 Department of Radiology, Kanagawa Cardiovascular and Respiratory Center, Yokohama, Japan; 4 Department of Radiology, Yokohama Rousai Hospital for Labour Welfare Corporation, Yokohama, Japan; 5 Department of Pathobiology, Yokohama City University Graduate School of Medicine, Yokohama, Japan; 6 Department of Respiratory Medicine, Tokyo Jikei University Hospital, Tokyo, Japan

**Keywords:** Systemic sclerosis, Emphysematous change, Vasculopathy

## Abstract

**Background:**

Pulmonary emphysema combined with systemic sclerosis (SSc)-associated interstitial lung disease (ILD) occurs more often in smokers but also in never-smokers. This study aimed to describe a new finding characterized by peculiar emphysematous change with SSc-associated ILD (SSc-ILD).

**Methods:**

We conducted a retrospective review of 21 consecutive patients with SSc-ILD diagnosed by surgical lung biopsy and focused on the radio-pathological correlation of the emphysematous change.

**Results:**

Pathological pulmonary emphysema (p-PE) with SSc-ILD was the predominant complication in 16 patients (76.2%) with/without a smoking history, of whom 62.5% were never-smokers. A low attenuation area (LAA) within interstitial abnormality on high-resolution computed tomography (HRCT) was present in 31.3%. Diffusing capacity of the lung for carbon monoxide (D_LCO_) was lower, disease extent on HRCT higher, and intimal/medial thickening in muscular pulmonary arteries more common in the patients with p-PE with SSc-ILD. However, forced vital capacity (FVC) was well preserved regardless of whether p-PE was observed. Most SSc-ILD patients had pulmonary microvasculature changes in arterioles (90.5%), venules (85.7%), and interlobular veins (81.0%).

**Conclusions:**

Pulmonary emphysematous changes (LAA within interstitial abnormalities on HRCT and destruction of fibrously thickened alveolar walls) are specific and novel radio-pathological features of SSc-ILD. Peripheral vasculopathy may help to destroy the fibrously thickened alveolar walls, resulting in emphysematous change in SSc-ILD.

## Background

Interstitial lung disease (ILD) is common in patients with systemic sclerosis (SSc), and a minority of patients evolve to end-stage respiratory insufficiency [1]. Ground-glass opacity, reticular intralobular interstitial thickening, and traction bronchiectasis are the most common findings on high-resolution computed tomography (HRCT) [2, 3]. The HRCT features of ILD in patients with SSc closely resemble those in patients with idiopathic nonspecific interstitial pneumonia (NSIP) [3]. In terms of pathological findings, NSIP is increasingly regarded as the more prevalent histologic pattern in patients with SSc-associated ILD (SSc-ILD) and in those with other connective tissue diseases (CTD) [4–8].

We previously reported that SSc-related autoantibody-positive ILD, but not CTD, is a different disease entity from that of SSc or mixed CTD associated with ILD [9]. In that report, pathological pulmonary emphysema (p-PE) with ILD was observed more frequently (56%) in the patients with SSc or mixed CTD associated with ILD. Interestingly, a high proportion (64.3%) of these patients were never-smokers [9], and another recent report found that pulmonary emphysema combined with SSc-ILD was also present in some never-smokers [10]. In contrast, Overbeek et al. attached importance to the correlation between vasculopathy and ILD in the patients with SSc [11]. Detailed radiological and pathological features of pulmonary emphysema combined with SSc-ILD have not yet been described. Therefore, this study aimed to clarify the clinico-radio-pathological characteristics of SSc-ILD, focusing on pulmonary emphysema and vasculopathy.

## Methods

This retrospective cohort study was approved by the institutional review board of Kanagawa Cardiovascular and Respiratory Center (no. 28–11). Because of the retrospective nature of the study, the review board waived the need for written informed consent from the patients.

The study population consisted of patients who had been diagnosed as having SSc-ILD, which was proven by surgical lung biopsy, at Kanagawa Cardiovascular and Respiratory Center between March 1997 and April 2016. The patients fulfilled the criteria for SSc of the American College of Rheumatology/European League Against Rheumatism classification [12]. One patient with SSc-rheumatoid arthritis overlap was excluded from this study, whereas 3 patients who developed manifestations of SSc during their follow-up period (0.42–2.17 years) were included. Baseline clinical measurements, radiological finding of HRCT, and right ventricular systolic pressure of echocardiography as indicator of pulmonary hypertension were obtained within 3 months of the histological diagnosis of ILD by surgical lung biopsy. The definition of chronic kidney disease were not a GFR below 60 mL/min/1.73m^2^ for three months or more or a GFR 60 mL/min/1.73m^2^ with kidney damage (i.e. marked of high levels of albumin in urine) [13]. Two radiologists (T. I. and S. I.) reviewed the HRCT scans for consensus of the diagnosis of ILD without knowledge of the patients’ clinical data. Patients were classified as presenting a HRCT pattern either “suggestive or consistent with NSIP” or “suggestive of usual interstitial pneumonia (UIP)” [14, 15]. The HRCT scans were analyzed for the following characteristics: honeycombing, ground-glass opacity, consolidation, reticulation, emphysema, cyst, traction bronchiectasis, bronchial wall thickening, mosaic attenuation, pulmonary artery dilation, enlarged mediastinal lymph nodes, micro-nodules, pleural thickening or effusion, and volume loss. These features were selected on the basis of previous studies [9, 16]. Moreover, low attenuation areas (LAA) within the interstitial abnormalities were also evaluated. This term was defined as low attenuation within an area such as a reticular shadow or traction bronchiectasis/bronchiolectasis, and the LAA itself had an apparent wall thickness of not more than 1 mm. Any disagreements between the two radiologists after the first assessment were resolved by discussion. The extent (%) of pulmonary abnormalities on HRCT was calculated with computer-aided 3D quantitative analysis [9, 17]. We also categorized the study patients by the easily applied disease staging of Goh’s criteria: l) limited disease or 2) extensive disease [18]. The surgical lung biopsy slides were reviewed by two pulmonary pathologists (K. O. and T. T.) who were blinded to the patients’ clinical and radiologic information. Histologic patterns were classified according to the current classification of idiopathic interstitial pneumonia [15]. The pathological features of lung parenchyma, airway, and pleural lesions were semi-quantitatively graded as 0 (absent), 1 (mild), 2 (moderate), or 3 (severe) [9, 19–21]. Pathological emphysema was defined as grade based on the number of alveolar pores due to alveolar wall destruction and the degree of enlarged alveolar lumina [22]. “Cicatricial emphysema,” defined as permanent enlargement of air spaces distal to terminal bronchioles caused by severe fibrosis (e.g. idiopathic pulmonary fibrosis), was excluded from the definition of emphysema [23]. Pulmonary vasculopathies, which include 1) muscular pulmonary arteries (from 200 to 300 μm in diameter) by the Heath-Edwards scoring system [24], 2) cellular and/or fibrotic intimal thickening and muscularisation in the interlobular veins and venules, and 3) changes such as medial hypertrophy and/or intimal thickening in the arterioles along the alveolar ducts (< 100 μm in diameter), were also evaluated based on previous reports [11, 25]. Any disagreements between the two pathologists were discussed until consensus was reached.

### Data analysis

Categorical baseline characteristics are summarized by frequency and percentage, and continuous characteristic are reported as mean ± SD. To detect differences between groups, the Wilcoxon test, Fisher’s exact test, or Mann-Whitney U test was used as appropriate. We considered *P* < 0.05 to represent statistical significance. All data were analyzed with SPSS version 22.0 (IBM Japan, Tokyo, Japan).

## Results

### Patient characteristics

Clinical characteristics of the 21 patients with SSc-ILD are summarized in Table 1. The proportion of patients who had smoke was 38.1%, and 3 patients (14.3%) had Sjögren syndrome. The median follow-up period was 2.44 years, and only 2 patients (9.5%) died during this period.

**Table 1:** Patient characteristics at surgical lung biopsy between the existence and non-existence of pathological emphysema with SSc-associated ILD

Characteristics	All subjects	Existence of pathological pulmonary emphysema		*P* value
		Yes (p-PE with SSc-ILD)	No	
No. of patients	21	16	5	
Female N (%)	18 (85.7)	14 (87.5)	4 (80.0)	> 0.999
Age, mean ± SD	60.3 ± 10.5	60.5 ± 10.5	59.8 ± 11.7	0.679
Never smoker N (%)	13 (61.9)	10 (62.5)	3 (60.0)	> 0.999
Sjögren’s syndrome N (%)	3 (14.3)	2 (12.5)	1 (20.0)	> 0.999
Digital ulcers N (%)	4 (19.0)	3 (18.8)	1 (20.0)	> 0.999
Hypertension N (%)	4 (19.0)	3 (18.8)	1 (20.0)	> 0.999
Chronic kidney disease N(%)	1 (4.8)	1 (6.3)	0 (0.0)	> 0.999
KL-6, U/mL	1350.5 ± 1042.8	1517.1 ± 1102.8	684.0 ± 255.1	0.089
SP-D, ng/mL	223.84 ± 132.42	214.86 ± 139.17	259.75 ± 109.89	0.395
Pulmonary function tests				
FEV_1_/FVC ratio, %	81.17 ± 5.95	80.67 ± 6.70	82.78 ± 2.02	0.741
FVC, % predicted	92.79 ± 16.76	90.52 ± 17.00	100.04 ± 15.33	0.215
D_LCO_, % predicted	69.85 ± 20.56	65.02 ± 20.11	87.98 ± 9.68	0.0163*
FVC/D_LCO_, ratio	1.41 ± 0.37	1.46 ± 0.39	1.22 ± 0.14	0.253
Disease extent on HRCT, %	24.93 ± 10.78	28.13 ± 10.56	15.35 ± 2.94	0.013*
RVSP on echocardiography (available N)	18	15	3	
RVSP, mmHg	33.3 ± 11.8	34.8 ± 12.5	26.2 ± 0.7	0.279
RVSP (< 35/ 35- < 50/ ≥50)	11/ 4/ 3	8/ 4/ 3	3/ 0/ 0	0.730
Staging				
Limited disease N (%)	13 (61.9)	8 (50.0)	5 (100.0)	0.114
Extensive disease N (%)	7 (33.3)	7 (43.7)	0 (0.0)	
Unknown N (%)	1 (8.3)	1 (6.3)	0 (0.0)	
Medication (during follow-up)				
PAH-specific drug therapy use^a^	2 (9.5)	1 (6.3)	1 (20.0)	0.429
Ca channel blocker or ACE inhibitor	5 (23.8)	4 (25.0)	1 (20.0)	> 0.999
Steroid use	10 (47.6)	9 (56.3)	1 (20.0)	0.311
Cyclophosphamide use	4 (19.0)	4 (25.0)	0 (0.0)	0.532
Cyclosporine, tacrolimus or azathioprine use	5 (23.8)	4 (25.0)	1 (20.0)	> 0.999
Pirfenidone or nintedanib use	3 (14.3)	3 (18.8)	0 (0.0)	0.549
Deaths N (%) (during follow-up)	2 (9.5)	2 (12.5)	0 (0.0)	
Median follow-up years (range)	2.44 (0.21–16.06)	2.23 (0.21–16.06)	5.07 (0.81–12.5)	

**Fig. 1:**
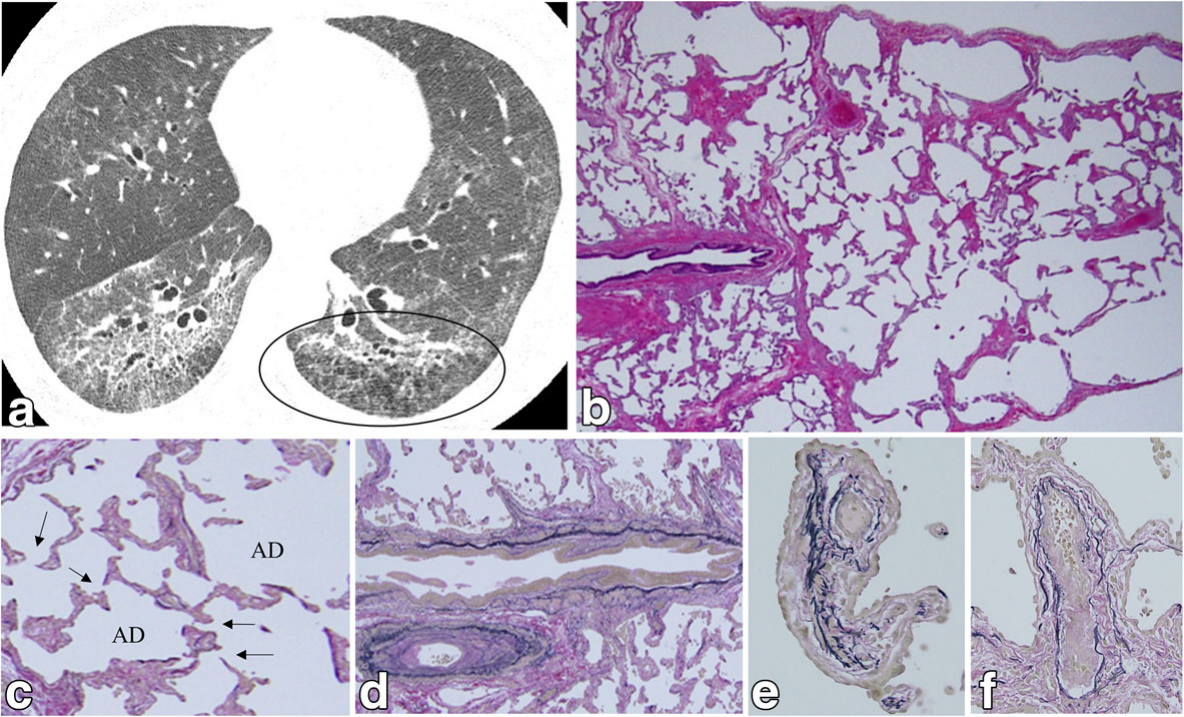
HRCT and surgical lung biopsy of a 45-year-old never-smoking woman. **a** Computed tomography scan shows traction bronchiectasis, reticulation predominantly in the peribronchovascular zone, and a low attenuation area (circle) in the subpleural area of both lower lungs. **b** Pathologic specimen (H & E staining, × 40) shows fibrotic nonspecific interstitial pneumonia with emphysematous change (grade 2). **c** At high-power magnification, emphysematous change occurs at the fibrously thickened alveolar walls showing an increase in pores (arrows) (Elastica van Gieson staining, × 100); AD: alveolar duct. **d** Muscular pulmonary artery shows intimal thickening (grade 1) (Elastica van Gieson staining, × 80). **e** Pulmonary arteriole lying along the alveolar ducts shows intimal thickening and muscularisation (Elastica van Gieson staining, × 200). **f** A venule shows intimal thickening and muscularisation (Elastica van Gieson staining, × 200)

**Fig. 2:**
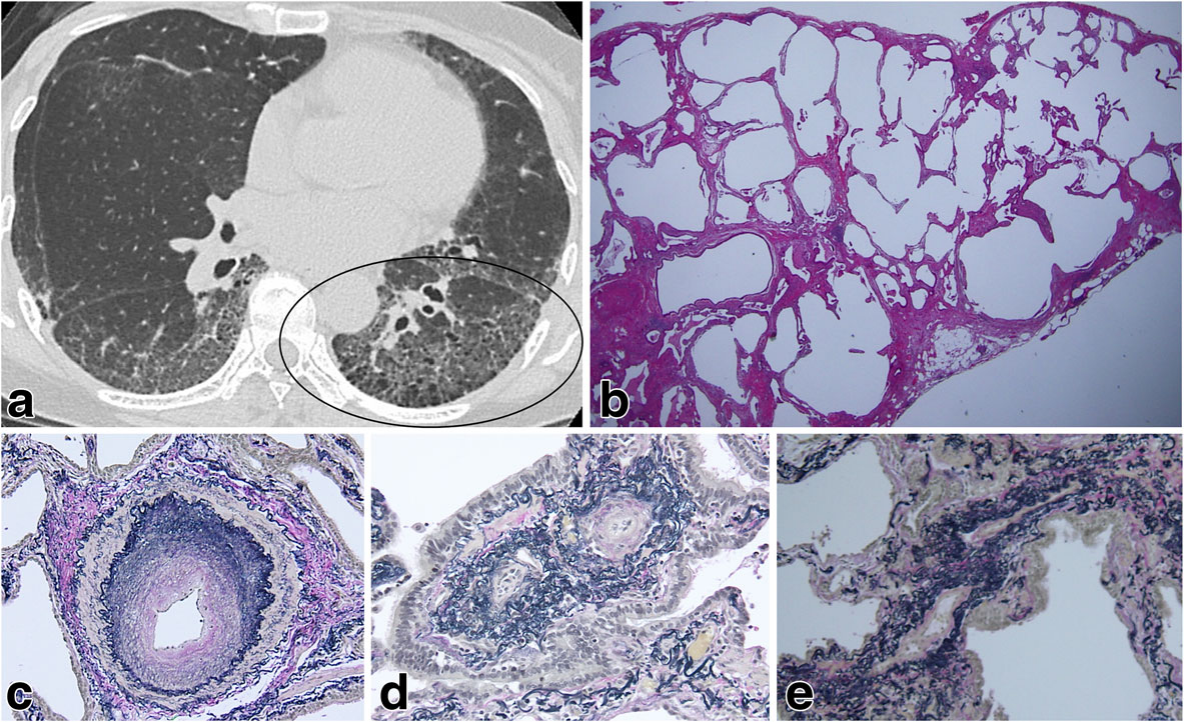
HRCT and surgical lung biopsy of a 70-year-old never-smoking woman. **a** Computed tomography shows reticulation and a low attenuation area within a ground glass opacity and reticulation (circle). **b** Pathologic specimen (H & E staining, × 40) shows fibrotic nonspecific interstitial pneumonia with emphysematous change (grade 1). **c** Vascular intimal and medial thickening of a muscular pulmonary artery (grade 2) (Elastica van Gieson staining, × 100). **d** Intimal fibrosis of alveolar interstitial vessels (arteriole) and **e**) (venule) (Elastica van Gieson staining, × 200)

**Fig. 3:**
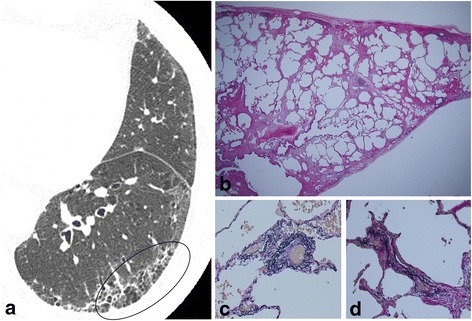
HRCT and surgical lung biopsy of 66-year-old ex-smoking man. **a** Computed tomography shows reticulation and a low attenuation area within the interstitial abnormalities (circle). **b** Pathologic specimen (H & E staining, × 40) shows fibrotic nonspecific interstitial pneumonia with emphysematous change (grade 2). **c** Intimal fibrosis of alveolar interstitial vessels (arteriole) and **d** (venule) (Elastica van Gieson staining × 200)

**Fig. 4:**
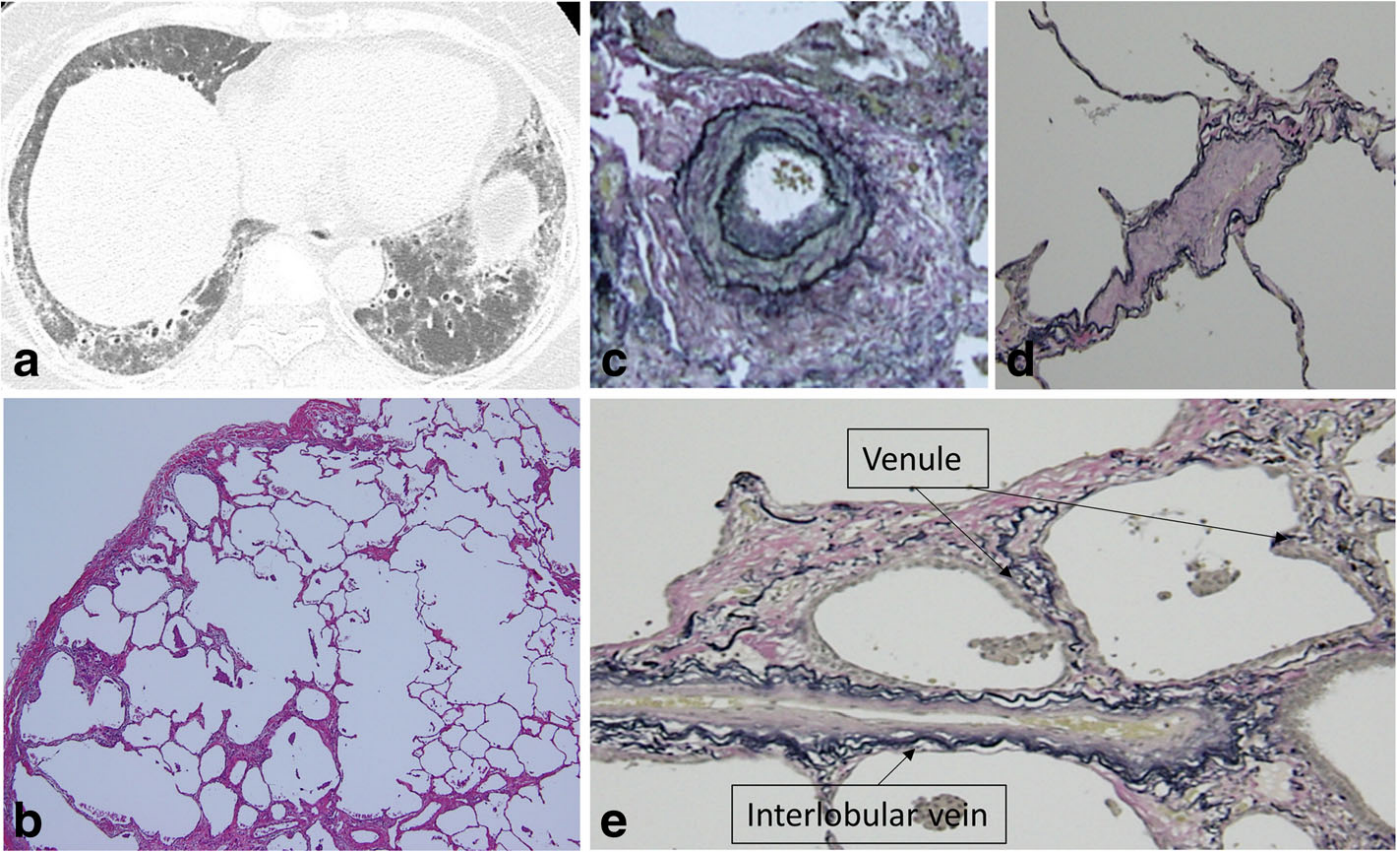
HRCT and surgical lung biopsy of 58-year-old never-smoking woman. **a** Computed tomography scan shows traction bronchiectasis, reticulation, and ground-glass opacity predominantly in the peribronchovascular zone of both lower lungs. **b** Pathologic specimen (H & E staining, × 40) shows fibrotic nonspecific interstitial pneumonia with emphysematous change (grade 2). **c** Muscular pulmonary artery shows medial hypertrophy (grade 1) (Elastica van Gieson staining, × 100). **d** Pulmonary venule lying along the alveolar ducts shows no apparent intimal thickening (Elastica van Gieson staining, × 200). **e** Venule and interlobular vein show intimal thickening (Elastica van Gieson staining, × 200)

Data are presented as mean ± SD, unless otherwise stated. *SSc* systemic sclerosis, *ILD* interstitial lung disease, *p-PE* pathological pulmonary emphysema, *KL-6* Krebs von den Lungen-6, *SP-D* surfactant protein-D, *FEV*_*1*_ forced expiratory volume in 1 s, *FVC* forced vital capacity, *D*_*LCO*_ diffusing capacity of the lung for carbon monoxide, *HRCT* high-resolution computed tomography, *RVSP* right ventricular systolic pressure, *PAH* pulmonary arterial hypertension. ^a^PAH-specific drugs include only beraprost sodium (*N* = 2). *P value less than 0.05

p-PE was observed in 16 patients (76.2%) of the patients with SSc-ILD. We classified the patients into two groups according to the existence or non-existence of p-PE with SSc-ILD. The proportion of never-smokers in the patients with p-PE with SSc-ILD was 62.5%. Smoking history for patients with each group was not significantly different. The diffusing capacity of the lung for carbon monoxide (D_LCO_) was significantly lower (*P* = 0.0163) and the disease extent on HRCT was significantly greater *(P* = 0.013) in the patients with p-PE with SSc-ILD. However, forced vital capacity (FVC) was well preserved in the patients regardless of whether p-PE was observed. The radiological and pathological characteristics of the patients are summarized in Tables 2, 3. The major HRCT pattern was NSIP in 19 patients (90.5%). The frequency of diffuse distribution of reticulation was more common in the patients with p-PE with SSc-ILD than in those without p-PE (*P* = 0.009). The association of vasculopathy with SSc-ILD patients are summarized in Table 4. LAA within the interstitial abnormalities was seen only in patients with p-PE with SSc-ILD (31.3%) (Figs. 1a, 2a, 3a, 4a). Fibrotic NSIP was the major histologic pattern seen in the SSc-ILD patients (85.7%). Of the SSc-ILD patients with p-PE, 12 (75.0%) had grade 1 emphysema, and 4 (25.0%) had grade 2 emphysema (Figs. 1b, 2b, 3b, 4b). Notably, emphysematous change in the SSc-ILD patients seemed to occur in the fibrotic alveolar walls and mainly presented as the destruction of fibrous thickened alveolar walls (Fig. 1c).

**Table 2:** Comparison of HRCT findings in patients with SSc-associated ILD

Characteristics	All subjects	Existence of pathological pulmonary emphysema		*P* value
		Yes (p-PE with SSc-ILD)	No	
HRCT pattern N (%)				
Suggestive of UIP	2 (9.5)	2 (12.5)	0 (0.0)	> 0.999
Suggestive or consistent with NSIP	19 (90.5)	14 (87.5)	5 (100.0)	
HRCT findings N (%)				
GGO	21 (100.0)	16 (100.0)	5 (100.0)	N/A
Consolidation	2 (9.5)	1 (6.3)	1 (20.0)	0.429
Reticulation	21 (100.0)	16 (100.0)	5 (100.0)	N/A
Honeycombing	1 (4.8)	1 (6.3)	0 (0.0)	> 0.999
Traction bronchiectasis	20 (95.2)	16 (100.0)	4 (80.0)	0.238
Bronchial wall thickening	10 (47.6)	7 (43.8)	3 (60.0)	0.635
Micro-nodules	3 (14.3)	3 (18.7)	0 (0.0)	0.549
Emphysema (LAA around no fibrotic appearance)	4 (19.0)	3 (18.7)	1 (20.0)	> 0.999
LAA within interstitial abnormalities	5 (23.8)	5 (31.3)	0 (0.0)	0.278
Cyst (non-honeycombing, emphysema)	5 (23.8)	4 (25.0)	1 (20.0)	> 0.999
Mosaic attenuation (air trapping)	7 (33.3)	4 (25.0)	3 (60.0)	0.280
Enlarged mediastinal lymph node	4 (19.0)	3 (18.7)	1 (20.0)	> 0.999
Pleural thickening or effusion	3 (14.3)	3 (18.7)	0 (0.0)	0.549
Pulmonary artery dilatation	6 (28.6)	5 (31.3)	1 (20.0)	> 0.999
Volume loss	17 (81.0)	14 (87.5)	3 (60.0)	0.228

Data are presented as mean ± SD, unless otherwise stated. *SSc* systemic sclerosis, *ILD* interstitial lung disease, *p-PE* pathological pulmonary emphysema, *HRCT* high-resolution computed tomography, *UIP* usual interstitial pneumonia, *NSIP* nonspecific interstitial pneumonia, *GGO* ground glass opacity

**Table 3:** Comparison of pathological findings (grade 0/1/2/3) in patients with SSc-associated ILD

Characteristics	All subjects	Existence of pathological pulmonary emphysema		*P* value
		Yes (p-PE with SSc-ILD)	No	
Lung parenchyma lesion				
Cellular infiltration	0/9/9/3	0/8/5/3	0/1/4/0	0.589
Lymphoid follicle with germinal center	12/7/1/1	10/5/0/1	2/2/1/0	0.349
Fibrosis	0/2/14/5	0/1/12/3	0/1/2/2	0.728
Honeycombing	15/5/1/0	12/3/1/0	3/2/0/0	0.601
Fibroblastic foci	4/16/1/0	3/12/1/0	1/4/0/0	0.781
Organizing pneumonia (intra-alveolar polyp)	11/8/2/0	8/7/1/0	3/1/1/0	0.927
Atelectasis (collapse)	0/11/5/5	0/10/4/2	0/1/1/3	0.046*
Cyst formation	14/5/2/0	11/3/2/0	3/2/0/0	0.882
Emphysema	5/12/4/0	0/12/4/0	5/0/0/0	< 0.001*
Pulmonary vascular change				
Muscular pulmonary artery	8/7/6/0	4/7/5/0	4/0/1/0	0.105
< none; grade 0/positive = grade 1–3>	8/13	4/12	4/1	0.047*
Arteriole <positive>	19 (90.5)	15 (93.8)	4 (80.0)	0.311
Venule <positive>	18 (85.7)	14 (87.5)	4 (80.0)	> 0.999
Interlobular vein <positive>	17 (81.0)	14 (87.5)	3 (60.0)	0.228

Data are presented as mean ± SD, unless otherwise stated. *SSc* systemic sclerosis, *ILD* interstitial lung disease, *p-PE* pathological pulmonary emphysema, *UIP* usual interstitial pneumonia, *NSIP* nonspecific interstitial pneumonia, *DIP* desquamative interstitial pneumonia. *P value less than 0.05

**Table 4:** Characteristics of the 21 patients with SSc-associated ILD

Patient	Sex	HRCT findings		Pathological findings		Pulmonary vascular change			
		Disease extent, %	LAA within the interstitial abnormalities	Classification	Emphysema, (grading score)	Muscular type, (grading score)	Arteriole	Venule	Interlobular vein
1	Female	41.37	+	fNSIP	2	1	+	+	+
2	Female	38.24	None	fNSIP	2	1	+	+	+
3	Female	42.37	+	fNSIP	1	2	+	+	+
4	Female	17.54	+	fNSIP	1	1	+	+	+
5	Female	13.65	None	Unclassifiable	1	1	+	+	+
6	Female	33.2	None	fNSIP	1	0	+	+	+
7	Female	13.47	None	fNSIP	1	0	+	+	–
8	Male	25.77	+	fNSIP	2	0	+	–	+
9	Female	18.86	None	fNSIP	1	0	+	+	+
10	Male	29.37	None	fNSIP	1	1	–	+	+
11	Female	N/A	None	fNSIP	1	2	+	+	+
12	Female	42.31	+	fNSIP	2	1	+	–	+
13	Female	16.7	None	fNSIP	1	1	+	+	–
14	Female	23.1	None	fNSIP	1	2	+	+	+
15	Female	36.22	None	Unclassifiable	1	2	+	+	+
16	Female	29.78	None	fNSIP	1	2	+	+	+
17	Female	19.58	None	fNSIP	0	0	+	+	+
18	Female	12.03	None	fNSIP	0	0	–	+	–
19	Male	13.3	None	fNSIP	0	2	+	–	+
20	Female	15.27	None	Unclassifiable	0	0	+	+	+
21	Female	16.55	None	fNSIP	0	0	+	+	–

*SSc* systemic sclerosis, *ILD* interstitial lung disease, *HRCT* high-resolution computed tomography, *LAA* low attenuation area, *fNSIP* fibrotic nonspecific interstitial pneumonia, *N/A* not applicable

Although the severity of changes in muscular pulmonary arteries in the patients with p-PE with SSc-ILD was not significantly different from that in the patients without p-PE (*P* = 0.105), its incidence was significantly higher (*P* = 0.047) in the patients with p-PE. Histological analysis found that 61.9% of the muscular pulmonary arteries were of Heath-Edwards grade 1–2 (Figs. 1d, 2c, 4c). Heath-Edwards grading of muscular pulmonary arteries of more than grade 2 (e.g. plexiform lesions) was not detected in our subjects. The incidences of changes in other pulmonary microvasculature were as follows: 90.5% showing arteriolar changes of intimal fibrosis and musculisation (Figs. 1e, 2d, 3c, 4d), 85.7% showing venular intimal thickening (Fig. 1f, 2e, 3d, 4e), and 81.0% showing intimal thickening of the interlobular veins (Fig. 4e). Most of the patients with SSc-ILD had arteriolar and venous-venular changes in their pulmonary microvasculature whether pathological emphysema coexist or not.

## Discussion

We investigated characteristic radiological and pathological features of 21 patients with SSc-ILD. This study presented that pulmonary emphysematous changes (LAA within interstitial abnormalities on HRCT and destruction of fibrously thickened alveolar walls) are specific and novel radio-pathological features of SSc-ILD. We provided two important findings. First, among patients with SSc-ILD, the complication of p-PE was frequently present in patients regardless of whether they had ever smoked. Surprisingly, 62.5% of the patients with p-PE with SSc-ILD were never-smokers. Historically, the syndrome of combined pulmonary fibrosis and emphysema (CPFE) has been individualized within the spectrum of smoking-induced chronic lung disease [26]. Later, Cottin et al. reported that the syndrome of CPFE warrants recognition as a novel distinct pulmonary manifestation within the spectrum of lung diseases associated with CTD, especially in patients with rheumatoid arthritis and SSc, whether current- or ex-smokers; however, 11.8% of their patients with CTD complicated by CPFE never smoked. [27] Antoniou et al. recently reported a prevalence of emphysema in patients with SSc-ILD of 12%; emphysema was present more often in current or former smokers, but it was also present in 7.5% of the never-smokers [10]. In both reports, although emphysema was analyzed as a radiological finding, the patients with CPFE had a lower D_LCO_, preserved FVC, which were also found in our study, and wider disease extent. However, their definition of CPFE was based on the presence of both emphysema predominant in the upper lobes and pulmonary fibrosis predominant in the lower lung zones [10, 27]. Because the emphysema in our study was analyzed pathologically, it is unclear whether these disease entities were evaluated from the same viewpoint in the previous studies. Our analysis emphasizes that the emphysematous change occurring with SSc-ILD is different from the usual form of smoking-related emphysema, which presents as destructive holes in the center of the secondary lobules and is generally well demarcated from the adjacent parenchyma and which also centers around the respiratory bronchioles and alveolar ducts [28]. In contrast, the emphysematous change in SSc-ILD mainly presents as destruction of fibrously thickened alveolar walls, resulting in abnormal dilatation of the alveolar lumina and alveolar ducts. Obviously, as mentioned above [23], “cicatricial emphysema” together with bronchiolectasis presents a characteristic gross appearance known as microscopic honeycombing that reflects end-stage fibrosis that is different from the emphysematous change in SSc-ILD. We believe that the presence of p-PE with SSc-ILD is more commonly seen in SSc patients than previously realized, even if they have never smoked, and that this is a new finding in SSc-ILD.

The second important finding was that LAA within interstitial abnormalities on HRCT might be a specific radiological finding in patients with SSc-ILD. Although LAAs are also shown on HRCT in a variety of fibrotic ILDs (in the extreme, honeycombing is composed of cystic spaces) [29], this finding refers to the fusing of discrete cysts with walls, whereas LAAs in our analysis were closely adjacent to interstitial abnormalities, and the LAAs themselves did not have an apparent wall thickness of more than 1 mm. This radiological finding was only seen in some of the patients with p-PE with SSc-ILD, and therefore, it might correlate with the pathological emphysema of SSc-ILD, though all our hypotheses were not corroborated.

In the vast majority of people, smoking is the cause of emphysema [30]. A previous radiological analysis showed that pulmonary microvascular blood flow is reduced in mild chronic obstructive pulmonary disease (COPD) [31]. In the aspects of the pathological analysis, pulmonary vascular changes in COPD or CPFE are characterized by a thickening of the vessel wall that begins early in the process of emphysematous change [25]. Thickening of the intima is the first structural change and is followed by an increase in smooth muscle cells and infiltration of the vessel wall by inflammatory cells [32]. In contrast, pulmonary vascular abnormalities are common in SSc patients, and the pulmonary hypertensive vascular changes manifest as concentric intimal thickening by fibromyxoid tissue and medial hypertrophy leading to thickened and stenotic pulmonary arterioles [33]. Most SSc patients with or without pulmonary artery hypertension (PAH) have intimal fibrosis and medial hypertrophy of the pulmonary arteries with an external diameter between 101 and 200 μm, which lie beside bronchioles [34]. Overbeek et al. reported that PAH in SSc patients is characterized by intimal fibrosis in the small vessels (arterioles, venules, and interlobular veins) [11]. Because changes were seen in 61.9% of the muscular pulmonary arteries in our patients with SSc-ILD, and in 90.5% of the arterioles and 85.7% of the venules in the microvasculature, these findings could be caused by reduced pulmonary microvascular blood flow, which might be one factor contributing to the emphysematous changes occurring in ILD.

Our study has several limitations. First, it was a single-institution, retrospective study with a small sample size. Second, there was selection bias because only the patients who could undergo surgical lung biopsy were included. Additionally, only two patients received PAH-specific drug therapy during the follow-up period, and most patients could not be evaluated for PAH by right heart catheterization. Therefore, more severe cases might present different radiological and pathological findings. Third, although this study reveals characteristic radio-pathological features in SSc-ILD, a comparable analysis was not performed for other etiologies of ILD (e.g. CTD-ILD). Fourth, although previous research showed that increased matrix metalloproteinases expression may play a role in accelerating the process of destruction as emphysema [35], immunohistochemical analysis could not be performed in our analysis and would be an issue in the future. Fifth, we used several types of HRCT scanning with the times. Therefore, imaging protocol may lead to a little different interpretation of radiological finding.

## Conclusions

Emphysematous changes, i.e. LAA within the interstitial abnormalities on HRCT and destruction of the fibrously thickened alveolar walls, are specific and novel radio-pathological features of SSc-ILD. Histopathological analysis suggested that peripheral vasculopathy may participate in the destruction of the fibrously thickened alveolar walls, resulting in the emphysematous changes seen in patients with SSc-ILD. Our results might help to provide more information for patients who do not fulfil the diagnosis of SSc (such as patients classified as having interstitial pneumonia with autoimmune features [36]) and suffer from a specific CTD diagnosis from the pulmonology viewpoint. Further studies are needed to clarify the quantitative loss of alveolar septa in the peripheral vascular bed that occurs with the emphysematous changes of SSc-ILD.

## Abbreviations

COPD: Chronic obstructive pulmonary disease
CPFE: Combined pulmonary emphysema with fibrosis
CTD: Connective tissue disease
D_LCO_: Diffusing capacity of the lung for carbon monoxide
FVC: Forced vital capacity
HRCT: High-resolution computed tomography
ILD: Interstitial lung disease
LAA: Low attenuation area
MCTD: Mixed connective tissue disease
NSIP: Nonspecific interstitial pneumonia
PAH: Pulmonary artery hypertension
PMBF: Pulmonary microvascular blood flow
p-PE: Pathological pulmonary emphysema
SSc: Systemic sclerosis
